# Direct imaging of intracellular RNA, DNA, and liquid–liquid phase separated membraneless organelles with Raman microspectroscopy

**DOI:** 10.1038/s42003-022-04342-4

**Published:** 2022-12-17

**Authors:** Ashok Zachariah Samuel, Kaori Sugiyama, Masahiro Ando, Haruko Takeyama

**Affiliations:** 1 Research Organization for Nano and Life Innovations, Waseda University, 513, Wasedatsurumaki-cho, Shinjuku-ku, Tokyo 162-0041 Japan; 2 Institute for Advanced Research of Biosystem Dynamics, Waseda Research Institute for Science and Engineering, Graduate School of Advanced Science and Engineering, Waseda University, 3-4-1 Okubo, Shinjuku-ku, Tokyo 169-8555 Japan; 3 Department of Life Science and Medical Bioscience, Waseda University, 2-2 Wakamatsu-cho, Shinjuku-ku, Tokyo 162-8480 Japan; 4 Computational Bio Big-Data Open Innovation Laboratory, AIST-Waseda University, Japan, 3-4-1 Okubo, Shinjuku-ku, Tokyo 169-8555 Japan

**Keywords:** Raman spectroscopy, Molecular imaging

## Abstract

Methodologies for direct intracellular imaging of RNA and DNA are necessary for the advancement of bioimaging. Here we show direct label-free imaging of RNA and DNA in single cells by isolating their accurate Raman spectra. Raman images of DNA from interphase cells show intact nucleus, while those from mitotic cells reveal condensed chromosome. The condensed chromosome images are accurate enough to assign the stage of mitotic cell division (e.g., metaphase). Raman spectral features indicate B-DNA double helical conformational form in all the cell lines investigated here. The Raman images of RNAs, on the other hand, reveal liquid-liquid phase separated (LLPS) membraneless organelles in interphase cells, which disappears during mitosis. Further, the Raman spectrum of proteins from the intracellular LLPS organelles indicates slight enrichment of amyloid-like secondary structural features. Vibrational imaging of intracellular DNA and RNA simultaneously would open myriad of opportunities for examining functional biochemical aspects of cells and organelles.

## Abstract (web summary)

Raman spectroscopy is employed to show simultaneous, label-free imaging of RNA, DNA, and proteins, revealing secondary structural features of intracellular LLPS organelles, and condensed chromosome.

## Introduction

Despite tremendous advancements in RNA technology, direct label-free imaging of RNAs (nucleic acids in general) in single cells remains a challenge. Fluorogenic RNA aptamers^1^, genetically engineered RNA molecules (MS2 loops) that binds to fluorescent proteins^2^, oligomeric fluorescent ligands bound RNAs etc.^3^ are used as labels in fluorescence technology to image intracellular RNAs. These strategies had revealed RNA-specific localization patterns in cells^2–4^. Alkyne-based Raman-tags were similarly employed in normal Raman and stimulated Raman scattering (SRS) microscopy to image distribution of biomolecules, including nucleic acids^5–7^. Imaging biomolecules in cells without external labeling is one of the prospects of Raman spectroscopy, but the technique had only limited success so far^8,9^. Differences in molecular structures of proteins, lipids, nucleic acids etc. give rise to clearly distinguishable molecular vibrational signatures in their Raman spectra. Using nonlinear coherent anti-Stokes Raman scattering (CARS), proteins and lipids were separately imaged in HeLa cells; however, due to highly overlapped nature of the spectra, RNA and DNA could not be imaged^10^. Nucleic acids specific wavenumber region in the SRS spectrum has been shown to have enough contrast to reveal nucleus^11^. Similarly, visualization of nucleus was also achieved with linear Raman spectroscopy and multivariate statistical analysis, however, the corresponding spectral component had mixed nucleic acid and proteins vibrational features^12^. For accurate imaging of intracellular DNA and RNA with Raman spectroscopy, the corresponding vibrational spectra should be accurately detected, which remains a challenge.

Recent advances in molecular biology have revealed functional membraneless organelle formation inside cells through liquid liquid phase separation (LLPS) of RNAs and proteins^13–15^. For instance, the formation of nucleolus through dynamic association of RNAs with other specific molecular units (e.g., rDNA, RNA polymerase etc.) for the synthesis of transcriptional system, *viz*., ribosomes, in eucaryotic cells^16^. Not only nucleoli but also several other nuclear aggregates, such as, Cajal bodies, nuclear speckles etc.^17^, each containing specific nuclear factors, form at different stages of cell cycle in a functional cell. Such dynamic associations of proteins and RNAs have now been recognized as a general mechanism by which cells function^18^. Proteins having intrinsically disordered regions (IDRs)^19^ with certain characteristic encoded sequences (i.e., prion-like)^20^ show high propensity to form LLPS domains in living cells. LLPS aggregates also play critical roles in disease progression^21,22^. Once the typically reversible LLPS aggregates^23,24^ phase-transition into a gel state—a matter of diagnostic importance—the process becomes irreversible^21^, which is a hallmark of diseases, such as, neurodegenerative diseases^24–26^. Secondary structural changes could occur in proteins and RNAs during LLPS formation^27^, but observing such structural modification inside cells is not an easy task. Despite considerable progress in LLPS research during the last decade^13,18,28,29^, investigating molecular characteristics of LLPS domains inside the crowded environment of cells remains a challenge^29^.

Raman spectra from cells and tissues serve as fingerprints of the specific crowded molecular environment, which can be used to classify them^30–33^. Quantitatively separating individual spectra of biomolecules, whose summation generates the composite Raman spectrum detected from each location of a cell, is a more challenging endeavor^12,33–35^. This latter approach is relatively easier in synthetic chemical systems and provides accurate quantification of molecules^36,37^. The success in biological systems, however, is often limited due to multiple reasons^12^, mainly highly fluctuating backgrounds and low signal to noise (SN) in the Raman spectral data^38^. Since RNA and DNA spectral components are relatively less pronounced in Raman spectra of cells, imaging them with Raman spectroscopy is particularly challenging. Our report, as far as we know, describes the first demonstration of non-invasive, simultaneous imaging of intracellular RNA and DNA without labeling. Spectral bands observed in the isolated Raman spectra of RNA and DNA were accurately assigned, which allowed retrieving secondary structural details of intracellular DNA, RNA, and proteins. Consequently, chromosome condensation, LLPS organelles, and several other molecular features of single cells are clearly revealed in our study.

## Results

**Fig. 1:**
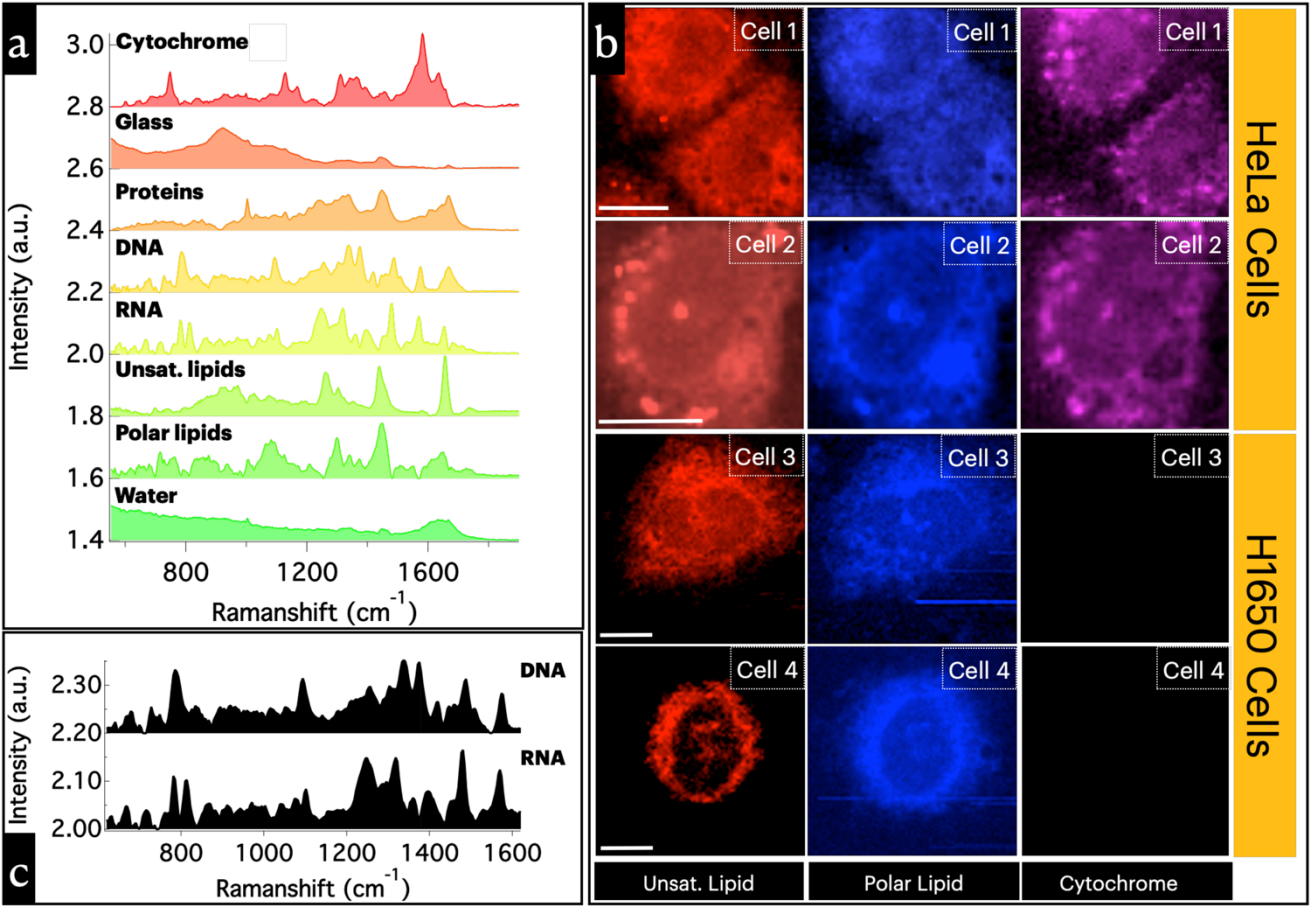
Results of multivariate data deconstruction using MCR-ALS. **a** MCR spectral components. **b** Raman images corresponding to cytochromes (***b*** and ***c***) and lipids in two different cell lines. Lipid droplet-like lipid enriched regions can be seen in Raman images corresponding to unsaturated lipids. **c** Raman spectral components corresponding to DNA and RNA (for details, see Supplementary Note 1 and Figs. S2–S8). Scale bars 10 µm.

In this study, we have investigated HeLa (cervical cancer cells), H1650 (Human lung cancer cell), and H1975 (Human lung cancer cell) with Raman micro-spectroscopic imaging. The spectra of individual biomolecular components were separated by applying multivariate curve resolution (MCR) analysis (Fig. S1). Standard-spectrum input was not used for performing the MCR analysis. Six spectral components (Fig. 1a) with molecularly assignable Raman spectral features of biomolecules were resolved in the MCR analysis (see “Methods”: Spectral assignments). The Raman images from HeLa and H1650 cell-lines corresponding to cytochromes and lipids are given in Fig. 1b. Polar lipids spectral profile had relatively lower SN, however, characteristics band near 720 cm^−1^ is clearly identifiable in addition to prominent alkyl chain, and ester linkage associated vibrational modes (see “Methods”: Spectral assignments).

Two spectral components in Fig. 1a, shown separately in Fig. 1c, require special attention. They are characteristically different from each other, and also from other biomolecules detected. These Raman spectral components could be accurately assigned to RNA^39^ and DNA^40^ (for details, see Supplementary Note 1 and Figs. S2–S8). Two peaks appearing at 783 and 813 cm^−1^ are Raman signatures of RNA^41^, whereas only one band appears for DNA at 785 cm^−1^ (cytosine ring breathing mode at 785 cm^−1^ and phosphodiester vibrational mode at 813 cm^−1^). DNA backbone PO_2_^−^ symmetric stretching vibrations can be seen at 1093 cm^−1^ ^40^, and the corresponding band for RNA appears in the region 1093–1101 cm^−1^ ^41^. The band in the region 1481–1487 cm^−1^ can be assigned to A/G ring stretching/planar vibrations, and the band at 1571–1576 cm^−1^ to A/G ring stretching modes of the nucleic acids^41^. The two broad bands centered at 1245 and 1318 cm^−1^ are characteristically strong in RNA spectra, which were assigned to U/C ring stretching A/G ring stretching, respectively^41^. Comparatively sharper bands are seen in the DNA spectrum at 1340 and 1375 cm^−1^, and a relatively weaker band at 1256 cm^−1^ ^40,42^.

### Interphase cells and LLPS organelles

**Fig. 2:**
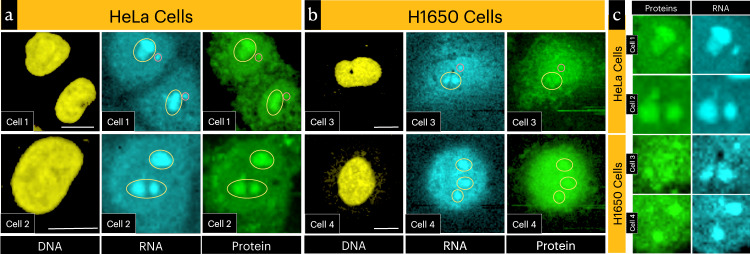
Raman images showing DNA, RNA, and proteins distributions inside cells. **a** HeLa cells, **b** H1650 cells. Nucleoli are circled yellow and Cajal bodies are circled red. A line profile from cell 2 is given in fig. S9. **c** Zoomed images from Fig. 2a, b showing RNA and proteins aggregates observed inside nuclear regions. Identical shapes confirm their assignments to nuclear LLPS domains. In cell 3, however, the LLPS image correlation is comparatively poor. Scale bars 10 µm.

Dense sub-nuclear domains of different sizes are generally observed in optical microscopy images of interphase cells. Fluorescence imaging after specific protein labeling revealed these domains as nucleoli and Cajal bodies^43^. Direct intracellular investigation of the molecular composition of these sub-nuclear aggregates requires simultaneous imaging of multiple chemical species. Previous Raman imaging studies failed to reveal the molecular architecture of such complex subnuclear features^5–9,11^. We examined whether the entire nuclear region has the same molecular constitution (e.g., DNA) or the dense regions are molecularly different. This information will help infer the molecular mechanism of subnuclear domain formation. The Raman images of RNA and DNA detected in our study are shown in Fig. 2. DNA (yellow) can be seen localized in the nuclear region (outside the nuclear region DNA spectral intensity is negligible)^11^, while RNA is distributed in both cytoplasmic and nuclear regions of the cells. The cellular distribution of RNA observed here agrees with fluorescence images reported earlier^10^. Interestingly, RNA Raman images show dense nuclear regions, but such domains are not seen in the DNA Raman images (Fig. 2). Therefore, these are clearly RNA aggregated domains. Importantly, Raman images of proteins also show dense domains of identical shapes (Fig. 2c), indicating that these are the LLPS organelles formed by the aggregation of proteins and RNAs. A simple simulation analysis indicated that H_RNA_ MCR profiles are not H_proteins_ duplication artifact (see “Methods”: Simulating spatial amplitudes and correlation analysis) Nucleoli in mammalian cells can reach a size of 3–4 µm while Cajal bodies can reach a size of about a micron^44^. From the number of LLPS observed inside the nucleus, their sizes, and based on previous studies^43–45^, we assign the observed larger (3–5 µm) LLPS structures as Nucleoli. The smaller ones in the range of 1 µm could be Cajal bodies (for detailed discussion, see Supplementary note 2)^43,44^. In cell 1 and cell 3 Cajal bodies can be identified (Fig. 2a; red circle) but not in other cells (Fig. 2). It is, however, known that a portion of HeLa cells in every culture invariably lacks Cajal bodies^46^ and in some cell-types they may even be completely absent or not observable^47,48^.

### Mitotic cells: condensed DNA and dissolution of LLPS organelles

Condensed chromosome is a characteristic feature of mitotic cells. Several protein molecules are implicated in the chromosome condensation process. For instance, the role of condensin complex in coiling chromatin fibers^49^. Similarly, heterochromatin segments are bundled into transcriptionally silent genomic foci by HP1α protein (specific interaction between post-translationally modified histone and HP1α)^50–52^. Therefore we asked: (a) is there detectable large-scale protein-DNA phase separation associated with chromosome condensation? If true, DNA and proteins images should show comparable condensed features in the corresponding Raman images, (b) does helical structure of DNA change during chromosome condensation? If it does, condensed chromosome region will give characteristically different DNA spectrum compared to interphase DNA. These aspects are discussed below.

**Fig. 3:**
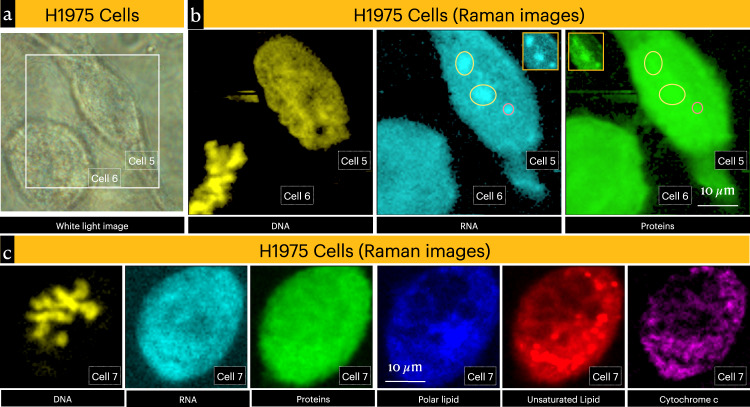
Raman images of mitotic cells. **a** White light image of elongated and circular H1975 cells, and **b** the corresponding Raman images of DNA, RNA, and proteins (*see* also Fig. S11). Nucleoli and Cajal body are marked with yellow and red circles, respectively. Inset shows better contrast Raman images of nucleoli and Cajal body from cell 5. Condensed chromosome (DNA) in circular cell (cell 6) indicates that they are in the metaphase of mitotic cell division. LLPS domains are absent in cell 6. **c** Raman images from another circular cell in the metaphase of mitotic cell division. LLPS organelles are also absent in the cell 7.

Cells undergo noticeable morphological change when they transition from interphase to mitotic stage. Among all the three cells-lines examined, H1975 cells showed easily identifiable circular and elongated cells in the same culture dish (Fig. 3a; see also Figs. S10 and S11). The Raman images from circular as well as elongated cells are shown in Fig. 3b, c. Condensed DNA can be clearly seen in circular cells, and from the chromosome morphology the cell is in the metaphase of mitosis^53^. It is also interesting to note that DNA spectrum from interphase cells and mitotic cells do not have large differences associated with helix modifications (Fig. S7). This suggests that chromosome condensation doesn’t involve major conformational reorganization of B-DNA double helix (premelting-associated differences are observed; Fig. S4). Further, we did not observe condensed protein (or RNA) domains in the mitotic cells. Proteins associated with condensed DNA may not have high enough concentration to generate contrast in the protein Raman images (Fig. 3)^54^. In other words, dense protein aggregates, similar to RNA-proteins in nucleoli, are absent at the condensed DNA domains. It is also important to point out that a large portion of HP1α has been shown to dissolve away from condensed DNA domains during mitosis^52^. A few other mitotic H1975 cells showing characteristically different DNA distributions (resembling late-prophase, prometaphase, and late anaphase) are shown in Fig. S10. However, it should be noted that a specific class of proteins (e.g., HP1α) inside cells cannot be selectively detected with single cell Raman imaging methodology unless there is a distinguishable molecular property (e.g., presence of heme in cytochrome).

Nuclear LLPS organelles form and disappear at different stages during the life cycle of cells. When interphase cells transition into mitosis, nuclear membrane breaks, and contents mix with cytoplasmic contents^55^. We have observed subnuclear domains in interphase cells (Fig. 2) assignable to nucleoli and Cajal bodies. Demonstrating disappearance of nucleoli and Cajal bodies during mitosis is necessary for proving accuracy of the methodology^23^. Simultaneous imaging of interphase and mitotic cell is important to confirm this aspect. Fortunately, we identified a mitotic cell and an interphase cell side-by-side in a culture dish. In the case of interphase cell (cell 5), nucleoli and Cajal body can be identified in the corresponding RNA and proteins Raman images. Importantly, sub-nuclear LLPS domains are absent in the corresponding Raman images from mitotic cells (cells 6 and 7)^23^. These results reaffirm the accuracy of our measurements and the assignment of LLPS domains.

### Secondary structural modifications of proteins in the LLPS domains

**Fig. 4:**
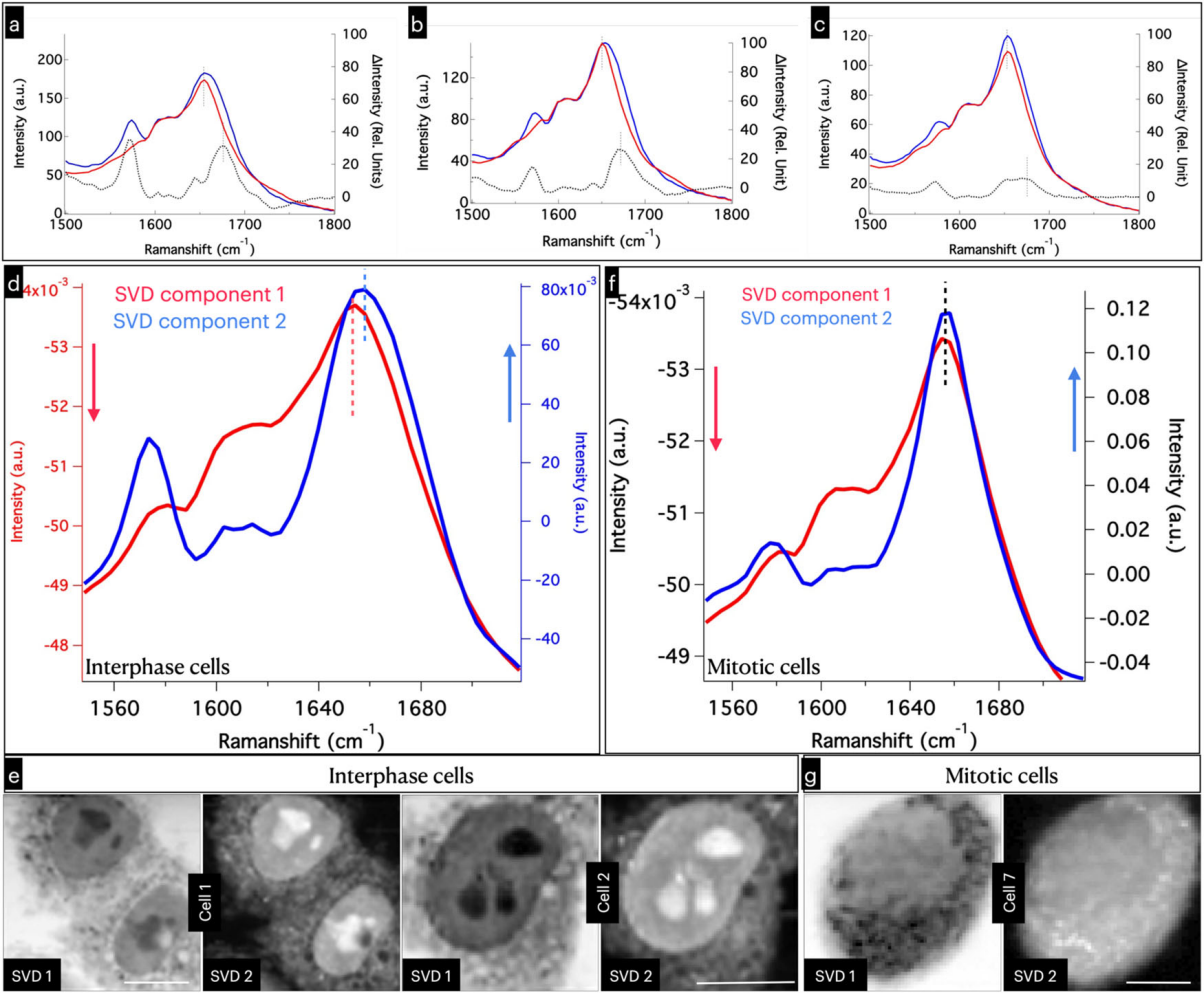
Analysis of amide I region of Raman spectra corresponding to proteins. Amide-I region of the average Raman spectra from LLPS (blue) and cytoplasmic regions (red). Strong 1573 cm^−1^ nucleic acids band can be seen. Amide I bandwidth changes are evident from the plots, which indicates changes in the secondary structure of proteins during LLPS aggregate formation (*See* also fig. S12). Black traces represent the corresponding difference spectra. **a** HeLa, **b** H1650, and **c** H1975. Results of SVD analyses (**d**–**g**). **d** First two SVD components from the interface cell Raman images. Amyloid-like spectral feature can be noted in SVD component 2 (Blue). **e** The corresponding interphase cell SVD images. **f** First two SVD components from the mitotic cell Raman image. Amyloid-like spectral feature is absent here. **g** The corresponding mitotic cell SVD images. Scale bar 10 µm. The full list of SVD spectra and SVD images are given in Fig. S13.

An important feature of Raman scattering is its sensitivity to molecular conformation. Amide-1 Raman spectral band appears in the 1600–1700 cm^−1^ wavenumber region, which has secondary structural details (helical, beta sheet, solenoid etc.) of proteins. While solving MCR, spectral components corresponding to conformationally different proteins should, in principle, be separated. But it is practically rather difficult to realize in the case of proteins without subjective errors because of the signal to noise (S/N) levels in the single-cell-Raman-spectra and broad nature of the band. However, since our study successfully identified LLPS organelles in the nucleus, the average spectrum of proteins in the LLPS domains can be compared to that of the cytoplasmic region. In the cells examined here, we have not detected LLPS organelles in the cytoplasm. Hence, we assume that the protein Raman spectrum from cytoplasmic region will have poorer LLPS associated secondary structural changes. Averaging large number of Raman spectra from multiple spatial regions also improves S/N permitting an accurate analysis of the amide regions. Water band (1650 cm^−1^) is relatively weaker and hence its influence is considered negligible as an approximation. Lipid band (1657 cm^−1^) is relatively sharper compared to amide-I band of proteins and where a change in bandwidth is not expected. Hence any change in the amide-I band in the average spectra can be assigned to secondary structural changes in proteins. The comparison of the amide-I region of the average Raman spectra of protein from LLPS and cytoplasmic region is shown in Fig. 4.

Characteristic differences between the structure of proteins (Φ and ψ angles of each amide bond^56^ and H-bonding differences) occupying LLPS organelles and cytoplasmic region is evident from the Fig. 4. The 1600–1635 cm^−1^ region of the amide band has been assigned to amino acid side-chain vibrations and aromatic amino acid ring vibrations^57^. The region from 1640 cm^−1^ is sensitive to secondary structure of proteins: 1644–1650 cm^−1^ indicates random coil structure, and vibrations in the range 1655–1668 cm^−1^ indicates α-helix secondary structure^58^. The region 1670–1682 cm^−1^ has been assigned to β-sheet secondary structure^59^ and 1683–1685 cm^−1^ region to β-turn structural motifs^60^. Additional broad feature is seen at the higher wavenumber region of the amide-I Raman band of proteins from LLPS regions (Fig. 4). The difference spectrum shows a band centered at 1670 cm^−1^. The secondary structure of proteins is different in LLPS domains compared to the structure they adopt in cytoplasm. Based on the above band assignments, it appears that the LLPS domains are slightly richer in beta-sheet and β-turn (*see* also Fig. S12)^27^.

SVD analysis of interphase and mitotic cell Raman images were also conducted. Interestingly, the first and second SVD spectral components have clear amide I vibrational features. Fortunately, band distortions associated with complex mixing of spectra are not observed in the first two SVD components, particularly in the amide I range^35^. In the interphase cells, the second SVD component with stronger 1573 cm^−1^ nucleic acid band shows broader amide I band compared to the first (Fig. 4d). This feature, however, is absent in the mitotic cells (Fig. 4f). The SVD results support enhanced amyloid-like features in LLPS domains^35^.

## Discussion

Separating Raman spectra corresponding to different classes of biomolecules is extremely important in accomplishing meaningful label-free imaging. It also permits examining the conformational characteristics of the intracellular biomolecules directly in cells. There are three structurally different DNA types known from the previous studies, such as A, B, and Z DNA^61^. The structural parameters of DNA double helix can also be expected to change in response to changes in the intracellular environment, such as, specific protein binding^62^. Since chromosome 3D organization induced altered gene regulation has been shown to occur during cancer, associated DNA helix conformational changes can also be suspected^63^. However, DNA spectral bands at 677, 729, 749, and 785 cm^−1^ observed in the present study agrees well with B-DNA helical form, suggesting no specific enrichment of A or Z DNA helix in the cancer cell lines examined here. Moreover, based on the above-mentioned low-frequency nucleoside vibrational modes, C2’-endo/anti vibrational characteristics can be deduced for G, A, and T in the intracellular DNA (Supplementary Table 1)^61^. Further, GC content estimated from O-P-O vibrational band at 835 cm^−1^ in the Raman spectrum of DNA revealed an average value of 47 ± 5%, which is close to the expected value from human genome analysis (41%; Fig. S3). Furthermore, DNA Raman band position at 1576 cm^−1^ in the spectra obtained from the metaphase H1975 cells indicates smaller premelting associated DNA structural modifications compared to interphase cells (H-bonding and base stacking associated changes; Fig. S4).

Compared to conformational analysis of DNA, analysis of RNA secondary structural features is complicated by the possibility of having multiple RNA conformational forms. The broad spectral features at 1245 and 1318 cm^−1^ are indicative of such structural complexity associated with conformational ensembles of RNAs in the intracellular environment. Spectral deconvolution analysis of the broad band centered at 1245 cm^−1^ clearly shows the presence of 1263 cm^−1^ peak, which is an indication of structured RNA rather than random configuration (Fig. S6)^64^. The intensity ratio I_814_/I_1101_ approaching 1.64 can be used as an indicator of ordered RNA structure^61^. A value greater than 1.2 has been estimated from all the MCR-isolated Raman spectra of intracellular RNA (Fig. S6a). Vibrations at 668, 716, 783, and 813 cm^−1^ further suggests that the majority of RNAs adopt A-RNA like configuration (not necessarily double helical) inside the cells^61^. There are also RNA Raman signatures that indicate possible hairpin loop, but the evidence is not confirmatory (Fig. S6c).

We have then compared Raman spectrum of total-RNA isolated from cells and intracellular RNA spectra (reflects average conformational information). Small but considerable spectral differences, suggesting secondary structural modifications, were observed in the difference spectra (Fig. S8). This suggests RNA secondary structural modifications in the intracellular domains due to conformational flexibility or due to intermolecular interactions with proteins or other biomolecules. Such features, in principle, can also be separated from a single cell under appropriate conditions. This points the possibility of resolving different conformational ensembles of RNA using Raman-MCR method in future studies.

## Conclusions

We have successfully demonstrated the application of Raman-MCR method for direct intracellular imaging of RNAs and DNA with secondary structural details. Clearly assignable RNA and DNA spectral components have been separated in this study, which allowed imaging their intracellular distribution without labeling. Identical shapes of dense regions of RNAs and proteins in the corresponding Raman images suggest the presence of LLPS organelles. These domains dissolved during mitosis proving the accuracy of the assignments and that of the methodology. On the other hand, chromosome condensation during mitosis does not involve large scale protein-DNA phase segregation. The Raman images of DNA revealed condensed chromosome structures in mitotic cells^65^. Further, we show that considerable DNA helix modification does not occur in mitotic cells during chromosome condensation. Amyloid-type protein structure modification during LLPS formation^27^, although proposed, has not yet been supported with the direct intracellular data from LLPS domains. Considerable changes in amide-I vibration indicating slight enrichment of amyloid-type protein features in LLPS domains were detected in this study. We believe that once the LLPS aggregates phase transition into insoluble aggregates, these features will be severely enhanced (a matter of medical importance). Application of this methodology to investigate different LLPS structures induced in the cells is promising.

## Methods

### Cell culture

Human cancer cells of Hela (ATCC, CCL-2), H1650 (ATCC, CRL-5883), and H1975 (ATCC, CRL-5908) were commercially purchased. Cells were cultured in RPMI-1640 (Gibco, #12633012) medium with 10% (v/v) fetal bovine serum (FBS) and 1× antibiotic-antimycoticum (Gibco, #15240062). These cells were seeded in 3.5 cm collagen-coated glass bottom dish (Matsunami, D11134H), 0.5 × 105 cells/dish, and incubated in the medium for 2–3 days, and then in serum-free medium for 1 day. After the incubation, cells were washed with PBS and fixed with 4% paraformaldehyde (PFA) for 15 min. Then PFA was removed by washing with PBS.

### Raman spectroscopy

Raman microspectroscopic imaging measurements were carried out with a laboratory-built confocal Raman microspectrometer. A 532 nm output of a Nd:YAG laser (Compass 315 M; Coherent Inc., Santa Clara, CA, USA) was used as the laser source. The laser beam was focused with a 100× (1.4 NA) objective lens (Plan Apo VC; Nikon Corporation, Tokyo, Japan) mounted on an inverted microscope (ECLIPSE Ti; Nikon Corporation, Tokyo, Japan). The back-scattered Raman light was collected and measured with a spectrometer (MS3504i, 600 lines/mm; SOL Instruments, Ltd., Minsk, Republic of Belarus) and a CCD detector (Newton DU920-M; Andor Technology Plc., Antrim, UK). The laser power at the sample was 15–20 mW. A piezoelectric stage (custom-made; Physik Instrumente GmbH & Co. KG, Karlsruhe, Germany) was used to carry out Raman imaging (0.3 μm step size) with 1 s exposure for each Raman spectral acquisition. Spatial resolution of the instrument is ~0.7 µm. Spectra were externally calibrated using indene. Raman data analysis and detailed image processing were performed with codes written in *Igor Pro*. Image J was also used to plot Raman images.

### Multivariate curve resolution (MCR)

#### Theory

MCR by alternating least squares (ALS) is performed by solving Eq. 1. Raman data matrix (*A*_*m,n*_) can be decomposed into spectral components (*W*_*m,k*_), and their concentration profiles (*H*_*k,n*_)^12,38^.

1 $${{{A}}}_{{{m}},{{n}}}={{{W}}}_{{{m}},{{k}}}\,{{{H}}}_{{{k}},{{n}}}+{{E}}$$

*E* is the error (or residual). In the present case, *A*_*m,n*_ consists of n spectra from different spatial locations of the cells each with m wavenumber points. MCR-ALS is performed iteratively by minimizing the Frobenius norm ||*A* − *WH*||^2^ until negligible residual *E* results. Non-negativity constraints *W* ≥ 0 and *H* ≥ 0 are applied during the minimization procedure to obtain interpretable solutions because all the Raman spectral intensities and the concentrations are necessarily positive. The number of independent spectral components (*k*) needed to be guessed at first and later optimized. The number of independent spectral components in singular value decomposition (SVD) served as an initial guess. The number of dominant singular values gives the number of spectral components as an initial value of *k*. Sparser MCR solutions can be sought by introducing regularization schemes such as L1 norm (Lasso regression) or L2 norm (Ridge regression). L1 norm can be applied to *H* matrix or *W* matrix depending on situation.

Following steps are involved in the analysis method used for resolving RNA/DNA spectra appropriately.

- Selected spectra analysis: Spectra from selected region of the cells (background region, nucleus, cytoplasm, etc.) were separately analyzed. The output from such analysis were then used as spectral input during whole cell data analysis.
- Sequential background/spectra optimization: Our modified routine for selective optimization of spectra was then employed for final MCR solution^12^.
- Successive optimization in multiple cells: Optimized MCR solution obtained for one cell was then fed into the MCR cycle of data from another cell, which was then further optimized. This complex procedure is pictorially represented in Fig. S14. The solution obtained in the complex cycle of curve resolution was then finally optimized for each cell type by selectively relaxing spectral vectors stepwise^12^.

### Spectral assignments of cytochromes^34^, lipids^12^, and proteins^12^

Cytochromes: A sharp band at 748 cm^−1^ can be reliably assigned to assigned to pyrrole breathing mode ν_15_ in cytochrome c. Sharp bands at 1584, 1312, and 1129 cm^−1^ can also be assigned to v(CαCm), ∂(CmH), and v(CαN) of the heme group of cytochrome c. The Raman bands at 1312, 1364, and 1394 cm^−1^ are the marker band for cytochrome c, while 1303 and 1338 cm^−1^ indicates the presence of cytochrome b. MCR resolved spectral profile (Fig. 1) shows Raman bands corresponding to both b and c.

Lipids: A band at 1749 cm^−1^ is due to the ester linkage in lipids. The band at 1657 cm^−1^ is an indicator of unsaturation in lipids (C=C). A relatively broad band at 1440 cm^−1^ is due to –CH_2_– scissoring mode (alkyl chain), prominent bands at 1305 cm^−1^ (CH– bending), and a broad band 1080 cm^−1^ corresponding to C–C stretching (rich in gauche conformation) along the carbon chain are all characteristic of lipids. Polar lipids show additional bands in 720 cm^−1^ region corresponding to choline vibrational mode.

Proteins: Prominent protein Raman spectral features include strong amide-I (peptide backbone vibration) at 1665 cm^−1^, C–H deformation mode at 1450 cm^−1^, tryptophan Cα–H deformation at 1340 cm^−1^, amide III at 1240 cm^−1^, and phenylalanine at 1003 cm^−1^. All these prominent features can be seen in the MCR spectral component assigned to proteins. Details of broad amide-I vibration is discussed in the main text.

RNA, DNA vibrational mode assignments are briefly described in the main text, and details are provided in Supplementary Note 1. Water bending mode at 1650 cm^−1^ is clearly seen in the MCR separated water Raman spectrum (Fig. S1; 8th spectrum from top). A spectral component with broad band in the region from 800 to 1200 cm^−1^ is identifiable in the glass background spectrum (Fig. S1; 2nd spectrum from top).

Analyses of spectral band features of proteins (comparison with the Raman spectrum of bovine serum albumin (BSA)) and cytochromes are provided in Fig. S15.

### Statistics and reproducibility

Multiple frames were collected for selected cells’ Raman images. The spectra and images were reproducible (Fig. S16), indicating minimal or negligible laser induced damage during image acquisition. Multiple cells from different cell-lines at different stages of cell cycle were imaged to confirm the accuracy of the methodology. Reproducibility of RNA and DNA spectral profiles were confirmed by resolving these spectral components from separate data acquired from three different cell lines (Fig. S7).

### Simulating spatial amplitudes and correlation analysis

Verifying the extent unmixing in spectral component is relatively easier since spectra of several pure biomolecules are known (Fig. S15). However, it is difficult to verify the accuracy of the MCR intensity profiles (spatial amplitudes; *H*). Therefore, we have performed a simple simulation experiment to further show that RNA MCR profiles (*H*_RNA_) are not mere duplicates of that of the corresponding proteins (*H*_proteins_) as a possible artifact of the methodology. The steps involved in the simulations are the following (Condition: *H*_proteins_ > *H*_RNA_).

**Fig. 5:**
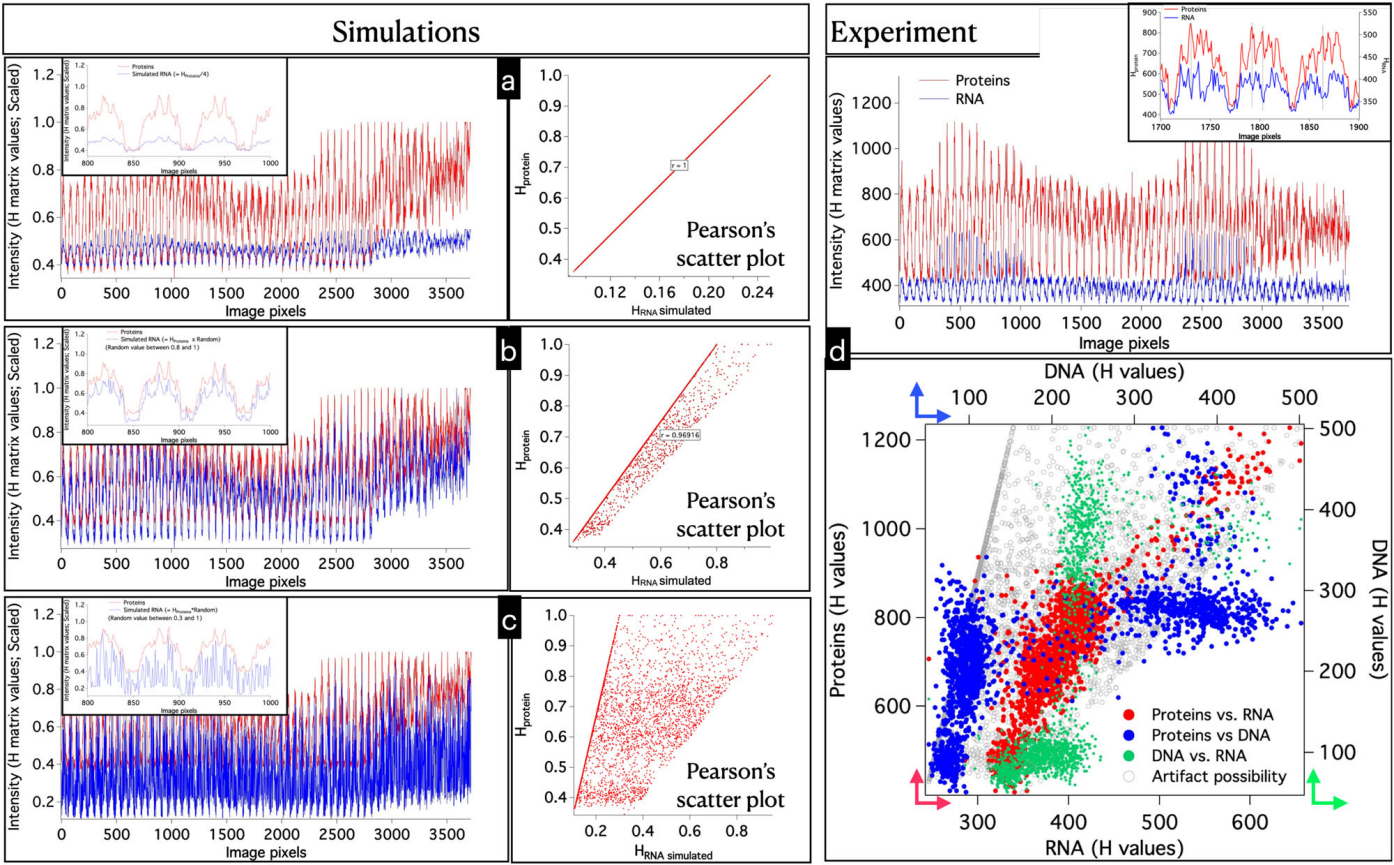
Correlation analysis of simulated and MCR resolved spatial amplitude (H) profiles. **a** *H*_RNA_ = *H*_proteins_/4; Pearson’s correlation coefficient = 1. **b** *H*_RNA_ = *H*_proteins_ × Random number (range 0.8–1.0); Pearson’s correlation coefficient = 0.96. **c** *H*_RNA_ = *H*_proteins_ × Random number (range 0.3–1.0); Pearson’s correlation coefficient = 0.44. The scatterplots for **b** and **c** show a characteristic trend: most of the points fall in a straight-line with other points scattered in a quadrilateral shape. **d** MCR resolved *H*_protein_ and *H*_RNA_ profiles. Pearson’s scatterplots for *H*_proteins_, *H*_DNA_, *H*_RNA_, and artifact possibility.

1. Obtain *H*_Protein_ values from the Raman image of an interphase cell.
2. Make *H*_RNA_ profiles by reducing the amplitude by fourth (*H*_RNA =_ *H*_Protein_/4).
3. Perform Pearson’s correlation analysis (Fig. 5a).
4. Generate random numbers for each image pixels.
  - Random numbers between 0.8 and 1.0 (Fig. 5b)
  - Random numbers between 0.3 and 1.0 (Fig. 5c)

5. Make *H*_RNA_ profiles by multiplying *H*_Protein_ by random numbers. (*H*_RNA =_ *H*_Protein_ × Random).
6. Perform correlation analysis for cases 4a and 4b.
7. Compare the results with the observed trend in MCR results (Fig. 5d).

The results of the analyses are provided in Fig. 5.

Pearson’s correlation coefficient ≠ 1 and the absence of the characteristic Pearson’s scatterplot indicates an acceptable *H*_RNA_ spatial profile. The observed *H*_RNA_ trend doesn’t fall into any of the artifact category. The observed H profile trend is unique for each biomolecule and is different from the artifact trend. This further confirms the acceptability of the analyses results.

### Reporting Summary

Further information on research design is available in the Nature Research Reporting Summary linked to this article.

## Supplementary information

Supplemental material

Description of Additional Supplementary Files

Supplementary Data 1

Reporting summary

## Data Availability

The data sets generated during and/or analyzed during the current study are available from the corresponding author on reasonable request. Source data for Fig. S3 (box plot) is provided in Supplementary Data 1.

## Code availability

Software implementing MCR-ALS can be found at https://github.com/mshrAndo/PyMCR.
