# Supplementary material for: Direct imaging of intracellular RNA, DNA, and liquid–liquid phase separated membraneless organelles with Raman microspectroscopy
Source: Commun Biol. 2022 Dec 17;5:1383. doi: 10.1038/s42003-022-04342-4 (PMC9759543; doi:10.1038/s42003-022-04342-4)
Supplement: Supplementary file 1 — Supplemental material [file 42003_2022_4342_MOESM1_ESM.pdf]

Supplementary Information

Direct imaging of intracellular RNA, DNA, and liquid-liquid phase separated membraneless organelles with Raman microspectroscopy

Ashok Zachariah Samuel,[1,*] Kaori Sugiyama,[2] Masahiro Ando,[1] Haruko Takeyama[*,1,2,3,4]

[1] *Research Organization for Nano and Life Innovations, Waseda University, 513, Wasedatsurumaki-cho, Shinjuku-ku, Tokyo 162-0041, Japan*

[2] *Institute for Advanced Research of Biosystem Dynamics, Waseda Research Institute for Science and Engineering, Graduate School of Advanced Science and Engineering, Waseda University, 3-4-1 Okubo, Shinjuku-ku, Tokyo 169-8555, Japan*

[3] *Computational Bio Big-Data Open Innovation Laboratory, AIST-Waseda University, Japan, 3-4-1 Okubo, Shinjuku-ku, Tokyo 169-8555, Japan*

[4] *Department of Life Science and Medical Bioscience, Waseda University, 2-2 Wakamatsu-cho, Shinjuku-ku, Tokyo 162-8480, Japan*

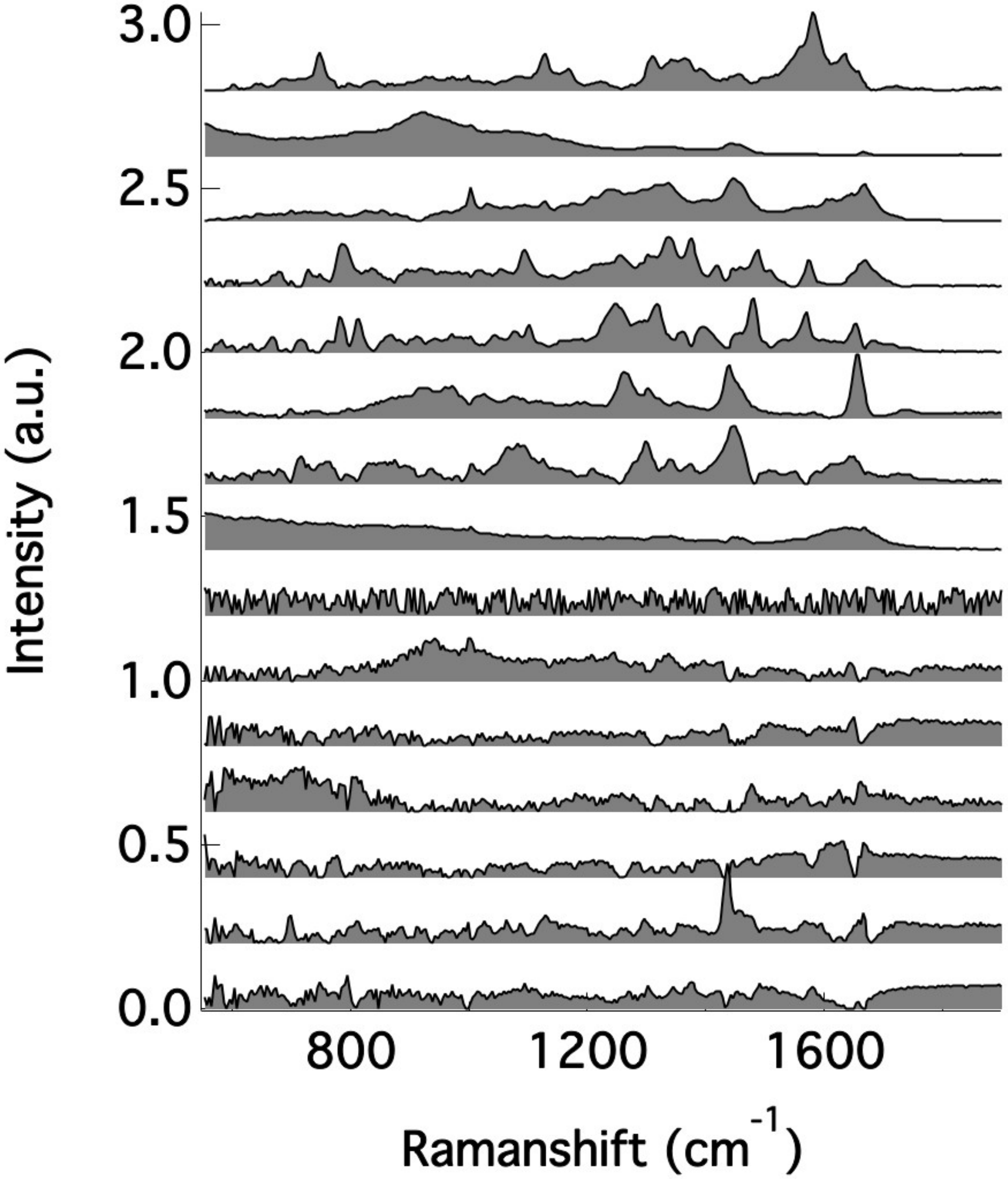

Fig. S1. List of 15 MCR spectral components. First 8 components have interpretable Raman spectral signatures. MCR component 14 shows 2 peaks which are not easy to assign and hence treated among the background components in the present study.

Supplementary Note 1

DNA and RNA: Band assignments and discussion

Earlier studies have noted that the observed vibrations of nucleic acids are localized group vibrations (backbone, sugars, and bases), and the mode coupling between them can be used to study changes in secondary structure of DNA and RNA.[1] Detailed analysis of the MCR derived RNA and DNA spectral components is necessary to confirm their accuracy, and to substantiate our claims of studying the secondary structure of DNA and RNA intracellularly. We have separated one RNA and one DNA spectra for each cell type. Small differences in the spectra from different cells should not be viewed as RNA/DNA differences between cell types. These are signatures representing 'frozen snapshot' of a collection of RNA/DNA molecules in different conformational state inside a very dynamic cell at the time of *fixing* (effects of PFA fixing is unknown now). Spectral features in the intracellular DNA/RNA spectral components are explained based on the existing knowledge from the studies on oligomeric forms and crystals of nucleic acids.

DNA band assignments and structural aspects of intracellular DNA

DNA has a double helical structure. Each sugar unit in each helix has one base connection and two phospho-ester connections. Sugar units in DNA conformationally reorganize depending on the overall structure of the helix. A unique set of seven dihedral angles can define one structural form. Any change in the helix is hence accompanied by changes in dihedral angles, that are manifested in vibrational frequencies. Phosphodiester modes in the range 700 to 850 cm^{-1} are sensitive to the dihedral angles of the O-P bonds.[1,2] The peaks observed in this region of the MCR isolated DNA spectrum is given in Fig. S2.

677 cm^{-1} has been assigned to G ring, 727 cm^{-1} to A ring, 749 cm^{-1} to T ring and 785 cm^{-1} to cytosine ring stretching vibrations (Table 1). Three different forms of DNA can be distinguished from vibrational signatures. Z-DNA gives identifiable O-P-O bands at 745 cm^{-1}, B-DNA at 835 cm^{-1}, and A-DNA at 807 cm^{-1}.[3] Among these phosphodiester modes representing different DNA conformers only one at 835 cm^{-1} is observed in MCR resolved DNA spectra. Therefore, it is very clear that the DNA observed in all the cell types are in B-DNA helical form. Interestingly,

depending on the base pairing (AT or GC) there are two groove dimensions (narrow and wide) in B-DNA. This makes 835 cm^{-1} O-P-O mode in B-DNA sensitive to the base pairing in the helical structure.[1] GC pair result in 830 cm^{-1} band, and AT pair generates a band at 840 cm^{-1}. The area intensity of these modes can be used to estimate GC content in the DNA (Fig. S3). An average GC content of 47±5 % was estimated, which is near the expected value of 41%.[1,3,4] However, it should be noted that several aspects, such as, cancer, can result in an increased GC content in human cells.[5]

The region from 1100-1600 cm^{-1} also shows clearly identifiable Raman bands. The results of band fitting analysis are shown in Fig. S4a. Band assignments are provided in the Table 1.[1–3] Based on the peak positions C2'-endo/anti dC , C3'-endo/anti dC and C2'-endo/anti dG, predominant C2'-endo/anti dA structural features can be inferred.[1,6] Sugar vibrational modes (850 to 1000 cm^{-1}) are relatively weaker and they are not clearly observed in the MCR resolved DNA spectra. The band around 1576 cm^{-1} (G and A ring vibrations) is sensitive to hydrogen bonding and base stacking. Based on variable temperature measurement conducted on model oligomeric DNAs, it has been shown that this mode is sensitive to premelting transitions in DNA.[1,6] Greater extent of 'premeltons' in DNA would cause the peak position to move to 1570 cm^{-1}.[7] The peak position at 1576 cm^{-1} for condensed DNA (mitotic) in H1975 cells thus indicates comparatively low levels of 'premeltons' (Fig. S4b).

RNA band assignments and structural aspects of intracellular RNA

RNAs can adopt different conformations in cells. However, like proteins, they are conformationally restricted and preferentially adopt energetically favorable structures.[8] Raman spectroscopy has been shown to be effective in distinguishing several structural aspects, such as, double helical, highly ordered single strand, and hairpin loops etc. Vibrational modes in the range 700 to 850 cm^{-1} are particularly useful in distinguishing RNA from DNA. Raman spectra of purified ribosomal RNA and transfer RNA have been reported.[9,10] All the spectral features observed are detected in the MCR separated RNA spectra. The results of band fitting analysis in the 700 to 850 cm^{-1} range are given in fig. S5a. Bands at 668, 716, 783, and 813 cm^{-1} agree with predominant A-RNA conformational form in cells.[11] Absence of 641 cm^{-1} band rules out Z-RNA

form (at least below detection limit). The band around 760 cm^{-1} can be seen as a shoulder in the RNA Raman spectra from HeLa and H1650 cells. However, this band is not very clear in H1975 cells (indicated with *). The phosphate vibration band shows predominantly one of the two different positions in different RNA spectra: in HeLa & H1975 (mitotic) this band appears at 1094 cm^{-1}, and in H1975 (interphase) and H1650 cells it appears at 1101 cm^{-1} (Fig. S5b). Origin of this change remains unclear.[12] However, apparently, the position of this band is sensitive to the base pair mismatch in RNAs.[13]

The intensity ratio of 813 and 1101 cm^{-1} Raman bands have been shown to depend on the number of phosphodiester linkages in *ordered* RNA configuration (like in double helical RNA, but not necessarily a double helix always).[3,12] A limiting value of ~1.64 was observed in earlier studies.[12] Estimate from our MCR resolved spectra are provided in fig. S6a. The band fitting parameters from fig. S5 were used in estimating relevant intensities, however, no baseline was used in estimating area intensities. In all the spectra, we observed value higher than 1.2 indicating ordered RNA structure and not random chain structure. However, metal ion binding of phosphate group can affect the intensity of phosphate stretching band.[14,15] Therefore, additional evidence is required to confirm the ordered structure of RNA inside cells. Variable temperature measurements on tetraloop hairpin forming RNA sequences indicated disappearance of 1263 cm^{-1} band at higher temperatures.[16] Absence of this band thus indicates *random* configuration of RNA. The broad spectral region from 1200 to 1280 cm^{-1} was resolved using reported band parameters[16] and the presence of this band has been verified (Fig. S6b**)**. Relative intensity of this band was the lowest in the RNA Raman spectrum from H1650 cell, in agreement with the corresponding lower I_{813} and I_{1101} ratio. Further, the band at 1570 cm^{-1} region[10] is known to shifts to higher frequency at higher temperature.[17] The band at 1570 cm^{-1} in RNA spectra again indicates ordered RNA structure. Further, by comparing the Raman spectra from hairpin loop forming RNA and poly-A.poly-U RNA, a band at 1595 cm^{-1} could indicate ordered hairpin loop structure in intracellular RNA (Fig. S6c).[16,17]

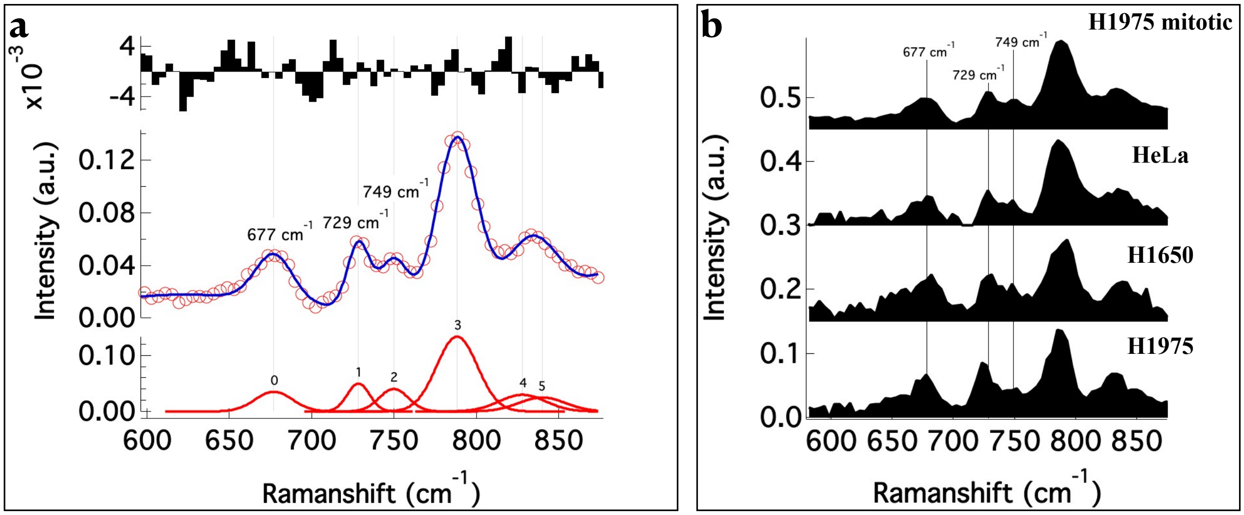

Fig. S2**.** a) Peak fitting analysis of MCR DNA spectrum. b) DNA spectrum isolated from different cell types. All the bands at 677, 729, 749, and 785 cm^{-1} can be identified in the spectral components. DNA spectrum isolated form the mitotic cell (H1975) has better signal to noise (SN) due to the condensed nature of DNA in the cell.

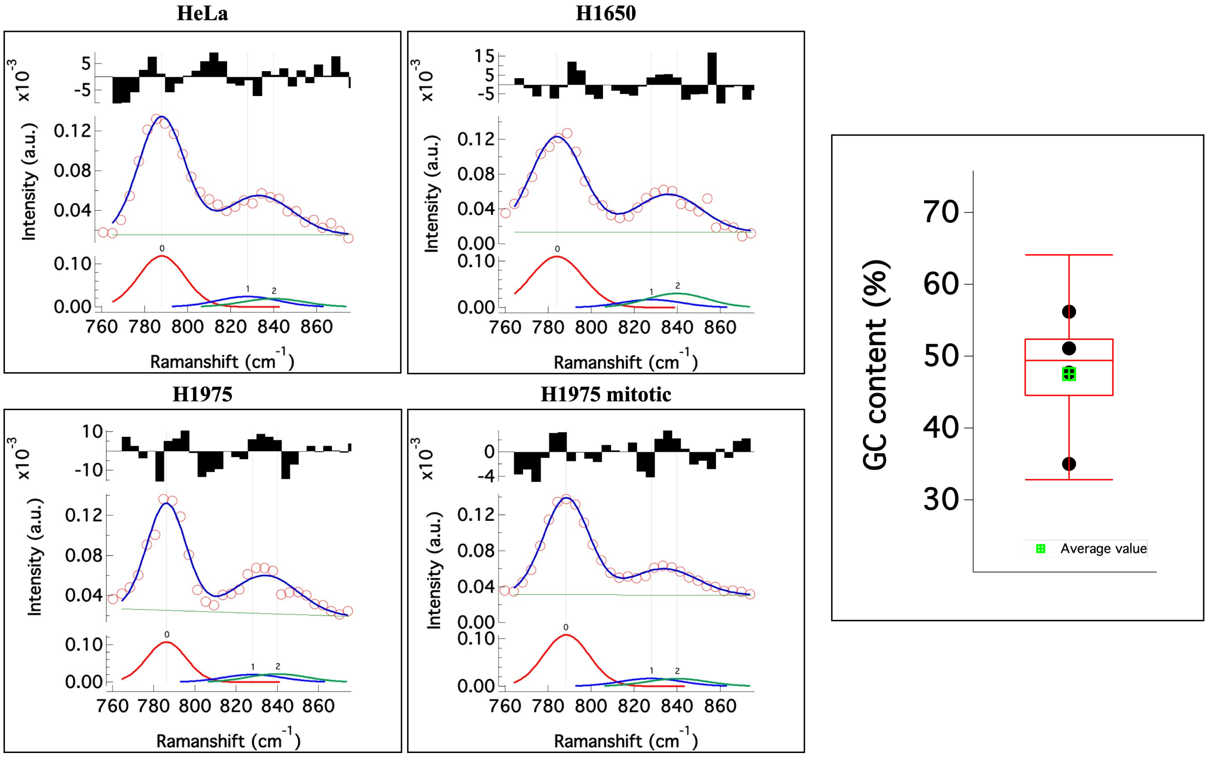

Fig. S3. Band fitting analysis of the O-P-O mode of B-DNA at 835 cm^{-1}. Area intensities of peaks 1 and 2 are used in estimating GC% (GC% = 100 $A_{GC}/(A_{GC}+A_{AT})$). GC% in human genome has been estimated earlier as 41%.[4] GC content estimated from different cells

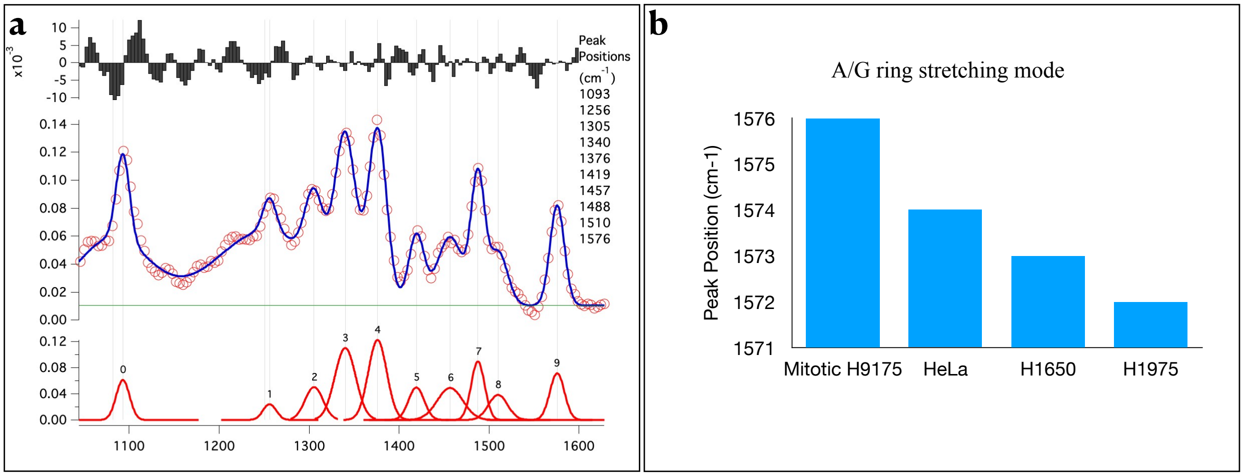

Fig. S4. a) Peak fitting results. b) Peak position of Raman band near 1576 cm^{-1}. Premelting of DNA results in lower band position.[1,6]

Supplementary Table 1. Assignments of Raman bands in MCR resolved DNA spectrum based on earlier studies.[1–3,6,9–12,14–17]

**RNA (cm^{-1})**	**Assignment**	**DNA (cm^{-1})**	**Assignment**
668	G	677	C2'-endo/anti dG , C3'-endo/anti dG[1]
710-719	C, A (705 for C and 718 for A, but the are not resolved in the MCR resolved RNA spectra)	727	C2'-endo/anti dA[2,17]
		749	C2'-endo/anti dT[1]
781-784	Cytosine ring breathing	785	Cytosine ring breathing
810-813	O-P-O (phosphodiester stretch)	830-840	O-P-O (phosphodiester stretch)
1093	PO_2^- symmetric stretch	1093	PO_2^- symmetric stretch
1101		1256	C2'-endo/anti dC , C3'-endo/anti dC

1245 (Broad)	C and U; Resolved into ~1224, ~1244 and ~1263 bands;	1340	A,G
1318	A, G; resolved into ~1294, ~1315, and ~1331 cm^{-1}	1375	T,A,G
1481	A, G	1420, 1457	dCH_2
1571	A, G	1487	A,G
1595	Unclear; may indicate tetraloop hairpin[16]	1570-1578	A, G (sensitive to premelting)
1646-1654	C=O	1661 (Broad)	C=O

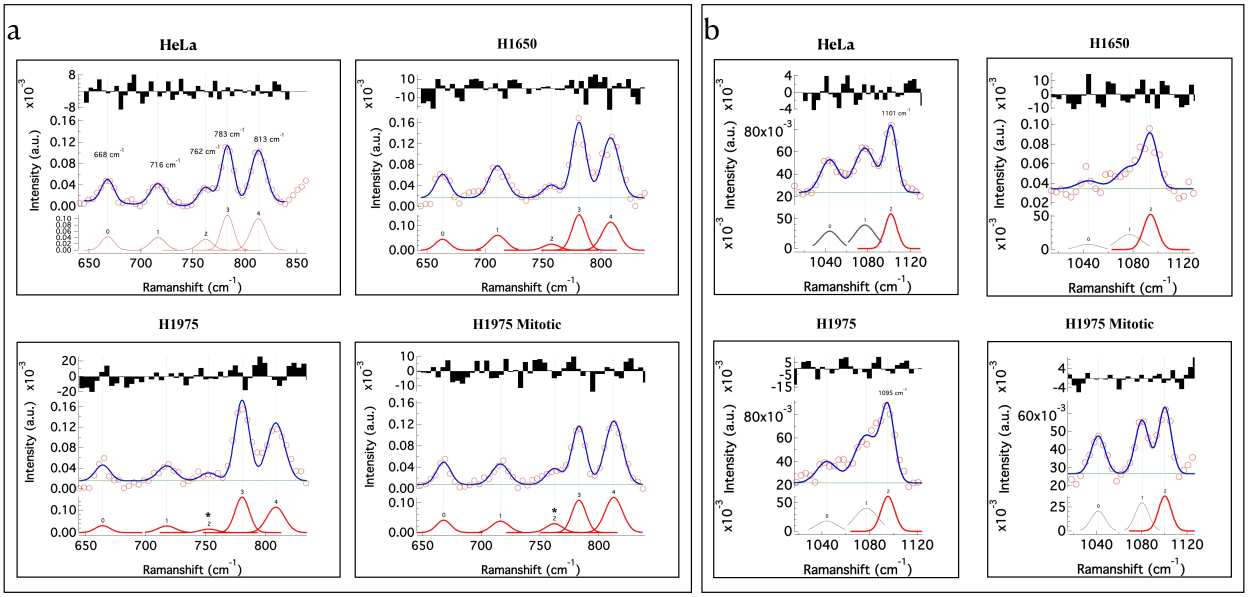

Fig. S5. Band fitting analysis of MCR resolved RNA spectra in the range a) 700 to 850 cm^{-1} and b) 1010 to 1140 cm^{-1}.

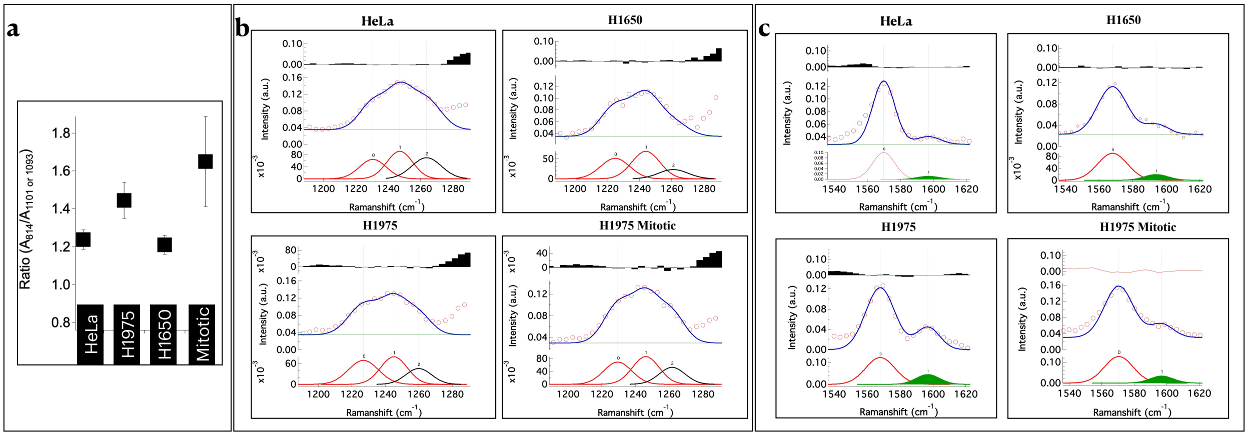

Fig. S6. a) Ratio of area intensities of 813 and 1101 (or 1094) cm^{-1} estimated from MCR resolved RNA Raman spectra. b) Band fitting in the region 1200 to 1280 cm^{-1}. c) band fitting in the region 1540 to 1620 cm^{-1} revealing a band at 1595 cm^{-1}.

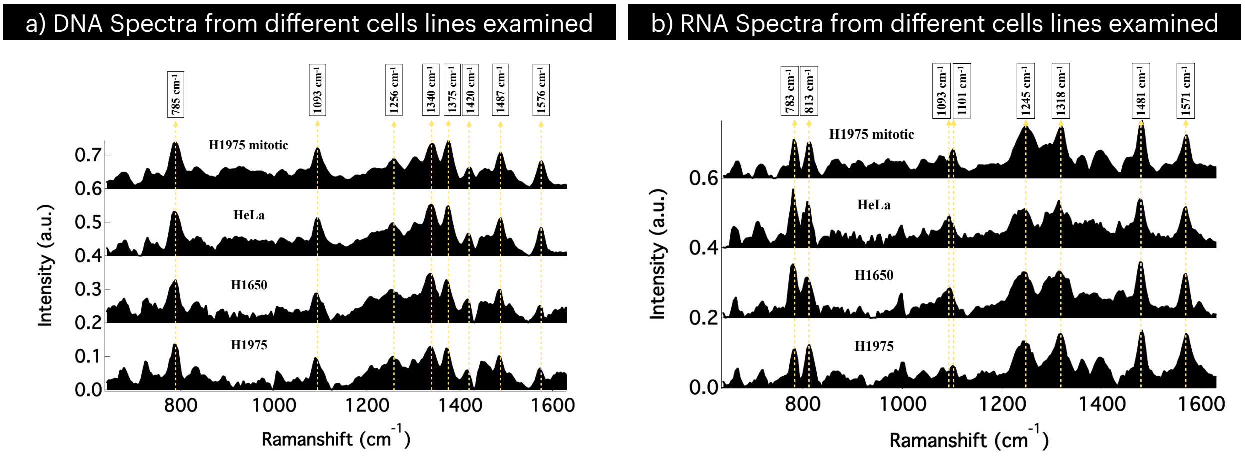

Fig. S7. DNA and RNA spectra obtained from different cell-lines examined in the study. DNA spectra are identical (within the limits of the analysis method), while RNA peaks show small differences in peak positions (PO_2^- symmetric stretching vibrational mode appears at 1093 and 1101 cm^{-1} for RNA from different experiments). Earlier studies have associated this peak shift with unusual backbone conformations of nucleotides.[13] All the main spectral features are reproducibly retrievable in the MCR analysis.

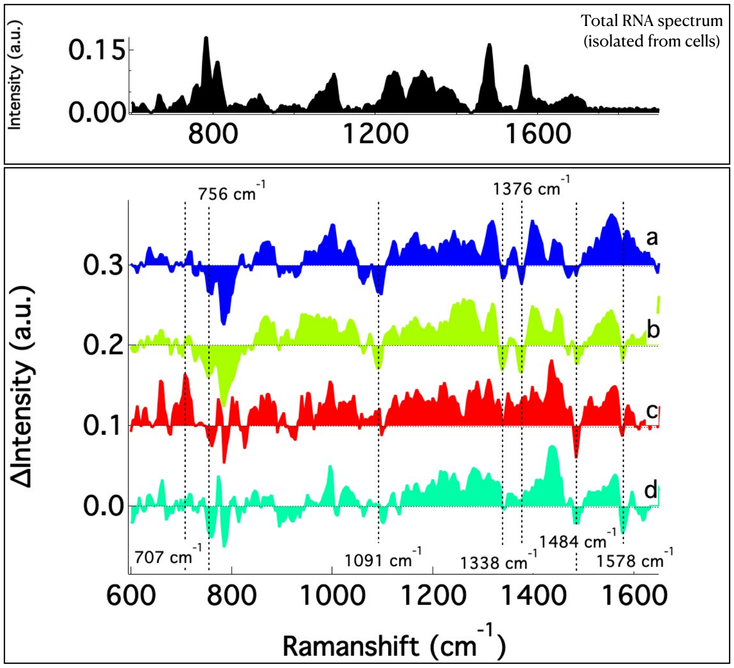

Fig. S8. Raman spectrum of Total RNA isolated from cells (Top). The difference spectra obtained by subtracting "total RNA" spectrum from intracellular RNA spectrum detected in, a) Mitotic H1975, b) HeLa, c) H1650, and d) H1975. Considerable differences are observed between the Raman spectra of intercellular RNA and that of isolated RNAs. Stronger difference intensities, we believe, are reflective of secondary structural differences. For instance, difference spectral band at 707 cm^{-1} in H1650 cell suggests possible conformational changes at C (and A) base units. Difference spectral bands at 1338 and 1376 cm^{-1} are suggestive of differences in RNA loop structures.[16] For more discussions, *see* supplementary note 1. RNA Isolation Kit: RNeasy Mini Kit – QIAGEN was used for isolating total RNA. The concentration of total RNA used for Raman measurement was ~30 µg/µL, which is close to the intracellular concentration of 68 pg/cell (~26 pg/pL).

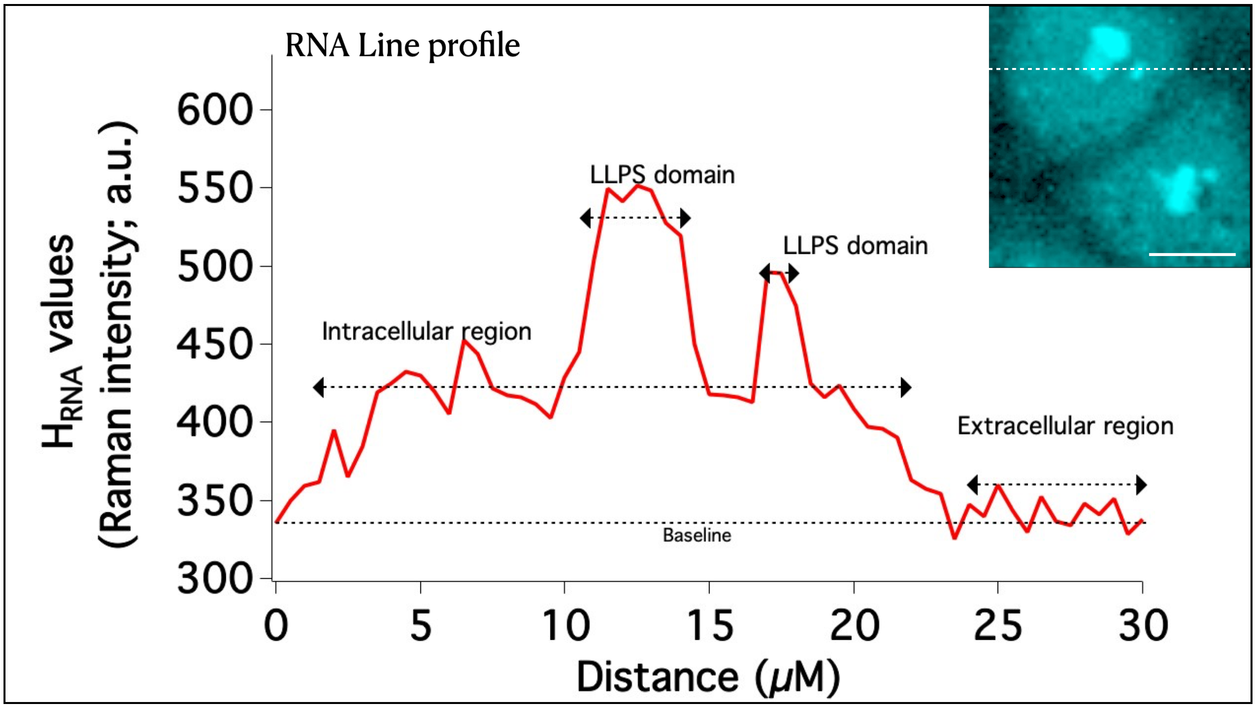

Fig. S9. A line profile from RNA Raman image of cell 2 (inset). Scale bar 10 μm.

Supplementary note 2

1. **PML bodies** are of similar sizes to that of Cajal bodies. PML bodies have been shown to associate with transcriptionally active regions. Their numbers are usually >10 in a cell.[18] We could observe one or two ~1 μm LLPS domains per cell. Importantly, they were absent in some cells. Transcriptionally active regions, we reasoned, should not be absent in a living cell. For the same reason we neglected the possibility of detecting **OPT domains**.[19] The PML aggregates may not be dense enough to generate concentration contrast in the Raman images, which could be the reason for not observing PML bodies.
2. **Nuclear speckles** are interchromatin granule clusters (rich in splicing factors), which consists of individual particles of size ~25 nm.[20] They can form local clusters with fine structure that can be resolved with structured illumination microscopy or super-resolution microscopy. Importantly they are scattered though out the nucleus of cells.[21] Under low resolution fluorescence microscopy, they can appear as a single domain. But their local density may not be adequate to give a concentration contrast, which is required for Raman imaging. Reviewer has also indicated this in the comments.

3. **Cleavage bodies:** These are not observed in interface cells.[22] Hence this possibility was excluded.
4. **Clastosomes** are special organelles produced when enhanced proteasomal activity is required (however not essential for proteasomal functions of a cell;).[23] They are usually rare in cells cultures under standard conditions.
5. **Cajal bodies**: Our justification for assigning the observed smaller nuclear aggregates as Cajal bodies are the following (in addition to size and number per nucleus). The contrast in Raman images comes from the density of the aggregated domains, which will enhance the intensity of Raman bands corresponding to constituent molecules. That is, nucleolus and Cajal bodies are denser compared to rest of the nucleus. Such denser bodies can give contrast even under whitelight microscopy.[24] Specific protein markers in these nuclear regions showing optical contrast have been confirmed in earlier studies: the smaller ones (~1 μm; Cajal bodies) contained coilin and the bigger one had fibrillarin (Nucleoli).[24] Hence, there is a high probability for observing these nuclear bodies in interface cells with Raman technique. Further, number, shape and sizes of observed aggregates also support our assignment (~1 μm LLPS domains were absent in many cells). Therefore, we believe that it is most probable that the smaller LLPS domains are Cajal bodies.

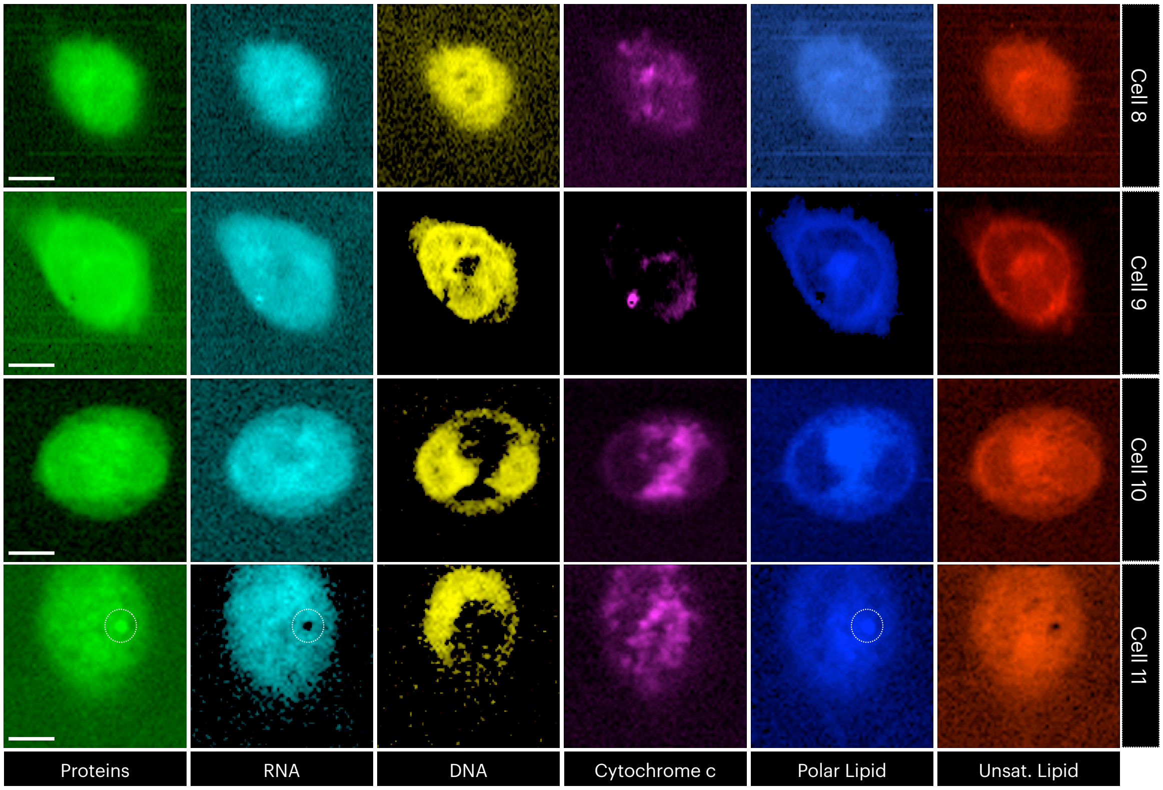

Fig. S10. Raman images of H1975 cells apparently under different stages of mitosis. Characteristically different DNA distributions can be clearly observed. RNA images of these circular/elliptical cells show no nuclear LLPS regions indicating mitotic stage. Based on DNA images cell 8 may be assigned to late-prophase, cell 9 to prometaphase, and cell 10 to late anaphase.[25] A peculiar distribution of DNA is seen in cell 11. Lipid aggregated domain (could be lipid droplet)[26] with proteins associated with it can be seen in cell 11. This region, however, lacks nucleic acids and hence are not LLPS domains. Scale bars 10 µm.

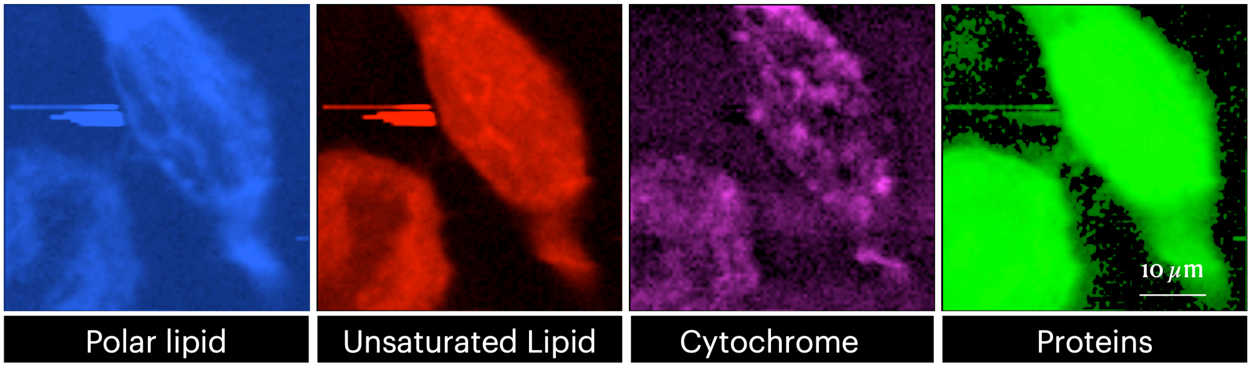

Fig. S11. Raman images from H1975 cells. RNA and DNA Raman images are given in fig. 3 in the main manuscript. Scale bar 10 μm.

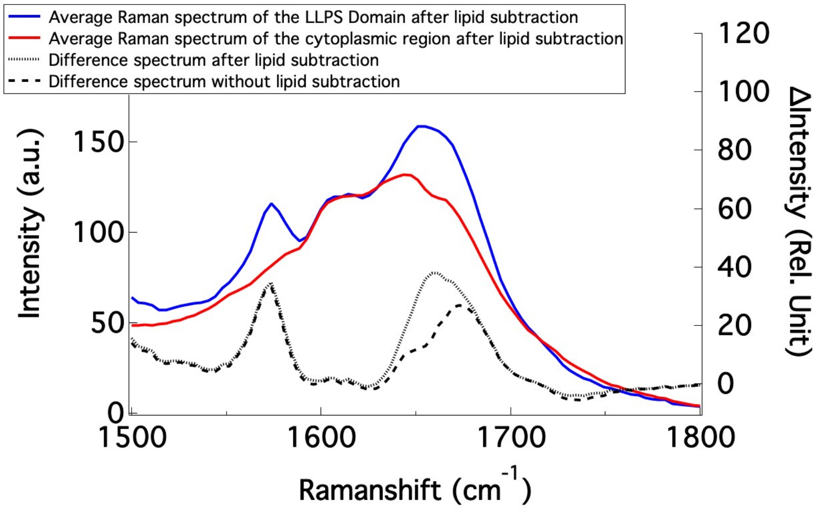

Fig. S12. Comparison of the average Raman spectra from LLPS domains and cytoplasmic region. Lipid contribution to these spectra were removed applying MCR results.[27] Blue, and red traces represent the average spectra from LLPS domain, and cytoplasmic region respectively. The black dotted line is the difference spectrum obtained after removal of lipid contribution from the average spectra. The black dashed line is the difference spectrum before subtracting the lipid contribution from the average spectra (*see* figure 4a, b, & c in the main manuscript).

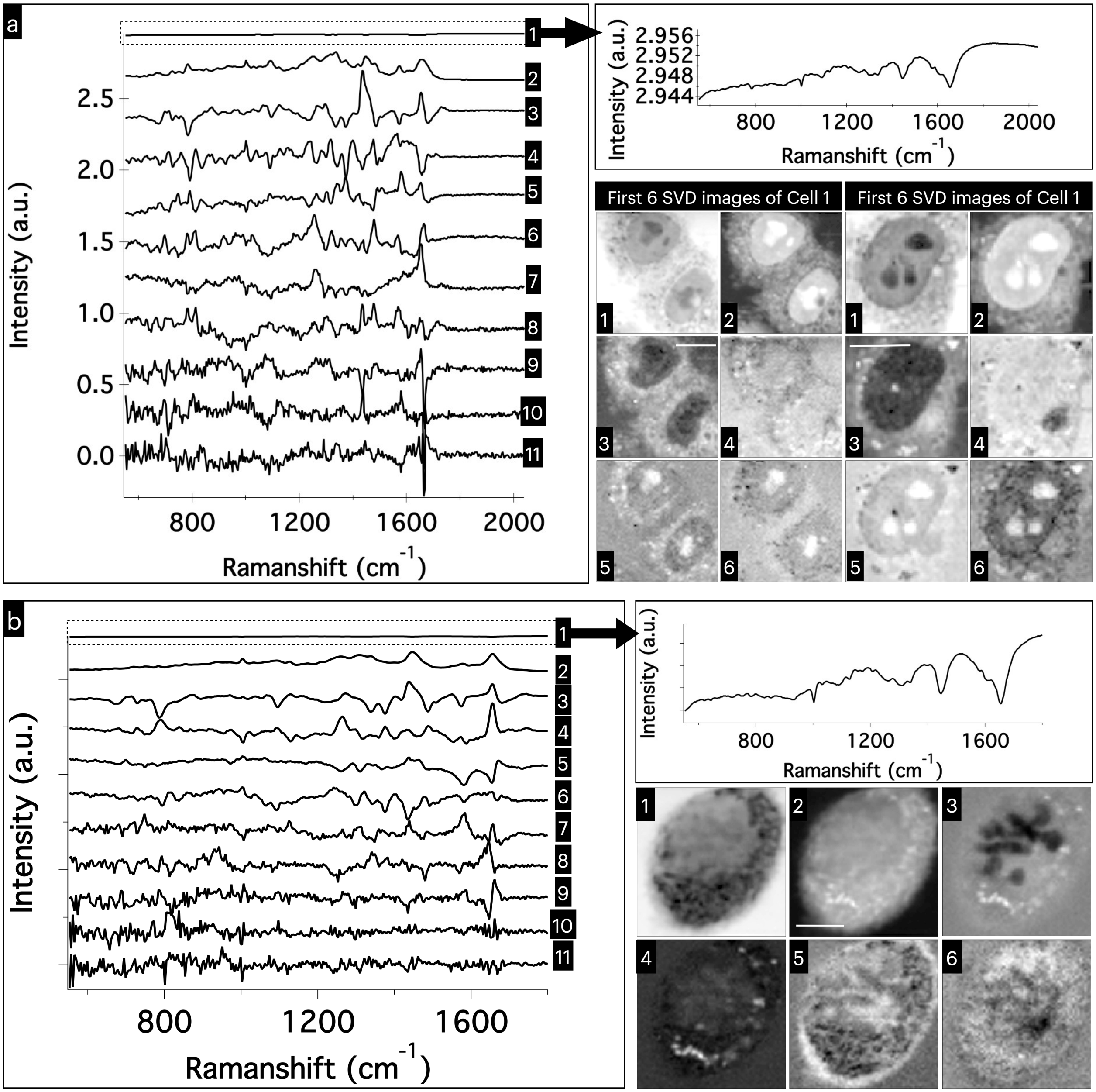

Fig. S13. Results of SVD analyses a) interphase cells b) mitotic cell. It is not easy to interpret the SVD images due to complex mixing of information.[28] Scale bar 10 µm.

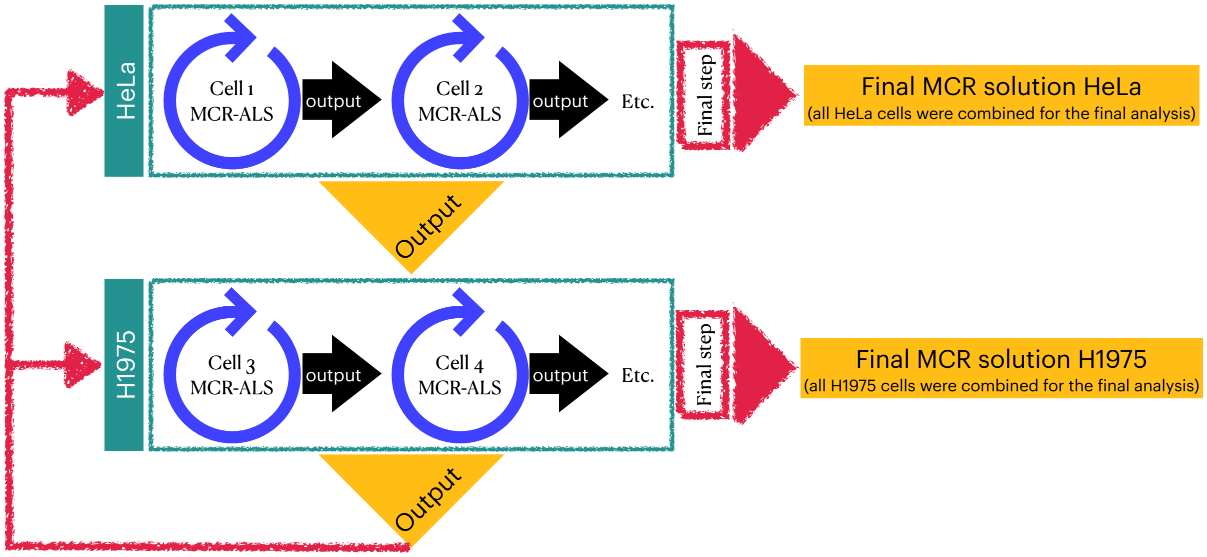

Fig. S14. Sequence of steps involved in performing the curve resolution. In the final step, combined data of all cells from each cell type was used as a single input matrix. Further, in the final step, the Frobenius norm was minimized by selectively relaxing spectral profiles and by simultaneously controlling L1 and L2 values. At the lowest possible minimum value of residual, all the constraints applied in the final step were removed and least square minimization was performed until satisfactory stable solution is obtained.

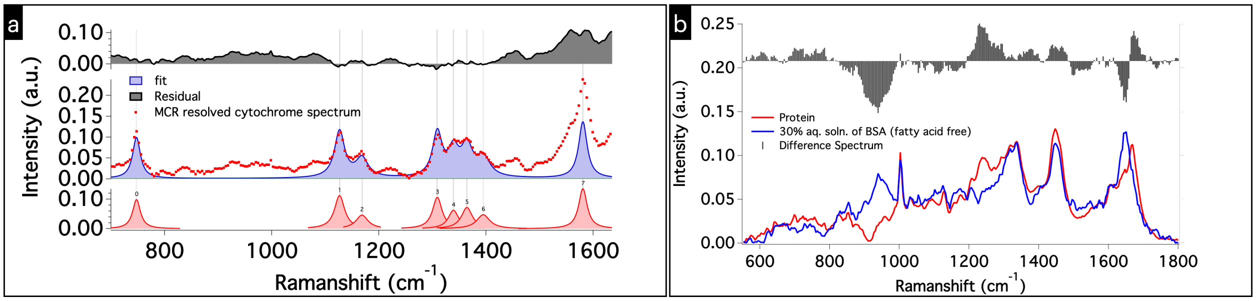

Fig. S15. a) Peak fitting analysis of MCR resolved cytochrome Raman spectrum. The peaks expected from heme unit of cytochrome are detected in the spectrum.[29] Residual has broad features 1600 cm^{-1}, which are hard to assign to any known chemical species. It is either characteristic feature of intracellular cytochrome or mathematical limitation of MCR. However, accuracy of the assignment is clear from the plot. b) comparison of the MCR resolved protein Raman spectrum with Raman spectrum of bovine serum albumin (BSA). These profiles show Pearson's correlation coefficient of 0.89. The observed differences are assignable: Broad feature at 938 cm^{-1} for BSA is characteristic of BSA aqueous solution indicating enhancement of alpha helical content,[30] which is also reflected in the BSA amide I band shift to lower wavenumbers. Broad amide III band at 1240 cm^{-1} is usually weak in BSA, while it is relatively stronger in MCR resolved protein as expected. Thus, the MCR spectral profiles resolved are clearly assignable.

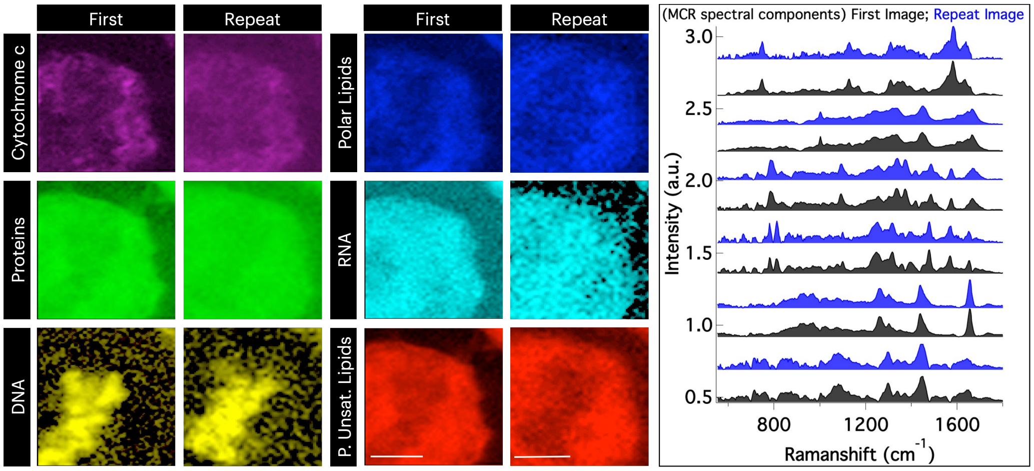

Fig. S16. Multiple frames of Raman images collected for cell 6. Scale bar 10 μm.

Supplementary References

1. Benevides, J. M., Overman, S. A. & Thomas, G. J. Raman, polarized Raman and ultraviolet resonance Raman spectroscopy of nucleic acids and their complexes. *J. Raman Spectrosc.* **36**, (2005).
2. Benevides, J. M., Wang, A. H. J., van der Marel, G. A., van Boom, J. H. & Thomas, G. J. Crystal and Solution Structures of the B-DNA Dodecamer d(CGCAAATTTGCG) Probed by Raman Spectroscopy: Heterogeneity in the Crystal Structure Does Not Persist in the Solution Structure. *Biochemistry* **27**, 931–938 (1988).
3. Eckstein, F. & Lilley, D. M. J. *Nucleic Acids and Molecular Biology*. (Springer, Berlin, Heidelberg, 1993). doi:https://doi.org/10.1007/978-3-642-77950-3.
4. Lander, E. S. *et al.* Initial sequencing and analysis of the human genome. *Nature* **409**, 860–921 (2001).
5. Watanabe, Y., Abe, T., Ikemura, T. & Maekawa, M. Relationships between replication timing and GC content of cancer-related genes on human chromosomes 11q and 21q. *Gene* **433**, 26–31 (2009).
6. Duguid, J. G., Bloomfield, V. A., Benevides, J. M. & Thomas, G. J. DNA melting investigated by differential scanning calorimetry and Raman spectroscopy. *Biophys. J.* **71**, 3350–3360 (1996).
7. Sobell, H. M. Premeltons in DNA. *J. Struct. Funct. Genomics* **17**, 17–31 (2016).
8. Schneider, B., Morávek, Z. & Berman, H. M. RNA conformational classes. *Nucleic Acids Res.* **32**, 1666–1677 (2004).
9. Thomas, G. J. Raman spectral studies of nucleic acids. III. Laser-excited spectra of ribosomal RNA. *BBA Sect. Nucleic Acids Protein Synth.* **213**, 417–423 (1970).
10. Tsuboi, M., Takahashi, S., Muraishi, S., Kajiura, T. & Nlshimura, S. Raman spectrum of a transfer RNA. *Science (80-.).* **174**, 1142–1144 (1971).
11. Cruz, P., Puglisi, J. D., Trulson, M. O., Tinoco, I. & Mathies, R. A. Raman Spectroscopic Study of Left-Handed Z-RNA. *Biochemistry* **26**, (1987).
12. Thomas, G. J. & Hartman, K. A. Raman studies of nucleic acids VIII estimation of RNA secondary structure from raman scattering by phosphate-group vibrations. *BBA Sect. Nucleic Acids Protein Synth.* **312**, (1973).
13. Hobro, A. J., Rouhi, M., Blanch, E. W. & Conn, G. L. Raman and Raman optical activity

(ROA) analysis of RNA structural motifs in Domain I of the EMCV IRES. *Nucleic Acids Res.* **35**, 1169–1177 (2007).

14. Medeiros, G. C. & Thomas, G. J. Raman studies of nucleic acids IV: Vibrational spectra and associative interactions of aqueous inosine derivatives. *Biochim. Biophys. Acta - Nucleic Acids Protein Synth.* **247**, 449–462 (1971).
15. Thomas, G. J., Medeiros, G. C. & Hartman, K. A. Raman studies of nucleic acids VI. Conformational structures of tRNAfMet, tRNAVal and tRNAPhe2. *Biochim. Biophys. Acta - Nucleic Acids Protein Synth.* **277**, 71–79 (1972).
16. Leulliot, N. *et al.* Unusual nucleotide conformations in GNRA and UNCG type tetraloop hairpins: Evidence from Raman markers assignments. *Nucleic Acids Res.* **27**, 1398–1404 (1999).
17. Lafleur, L., Rice, J. & Thomas, G. J. Raman studies of nucleic acids. VII. Poly A · poly U and poly G · Poly C. *Biopolymers* **11**, 2423–2437 (1972).
18. Wang, J. *et al.* Promyelocytic leukemia nuclear bodies associate with transcriptionally active genomic regions. *J. Cell Biol.* **164**, 515–526 (2004).
19. Harrigan, J. A. *et al.* Replication stress induces 53BP1-containing OPT domains in G1 cells. *J. Cell Biol.* **193**, 97–108 (2011).
20. Thiry, M. The interchromatin granules. *Histol. Histopathol.* **10**, 1035 (1995).
21. Wansink, D. G. *et al.* Fluorescent labeling of nascent RNA reveals transcription by RNA polymerase II in domains scattered throughout the nucleus. *J. Cell Biol.* **122**, 283–293 (1993).
22. Zeng, C., Kim, E., Warren, S. L. & Berget, S. M. Dynamic relocation of transcription and splicing factors dependent upon transcriptional activity. *EMBO J.* **16**, 1401–1412 (1997).
23. Lafarga, M. *et al.* Clastosome: A Subtype of Nuclear Body Enriched in 19S and 20S Proteasomes, Ubiquitin, and Protein Substrates of Proteasome. *Mol. Biol. Cell* **13**, 2771–2782 (2002).
24. Gall, J. G. The centennial of the Cajal body. *Nat. Rev. Mol. Cell Biol.* **4**, 975–980 (2003).
25. Paweletz, N. Walther Flemming: pioneer of mitosis research. *Nat. Rev. Mol. Cell Biol.* **2**, 72–75 (2001).
26. Samuel, A. Z. *et al.* Molecular profiling of lipid droplets inside HuH7 cells with Raman micro-spectroscopy. *Commun. Biol.* **3**, 372 (2020).

27. Samuel, A. Z. *et al.* Determination of Percent Crystallinity of Side-Chain Crystallized Alkylated-Dextran Derivatives with Raman Spectroscopy and Multivariate Curve Resolution. *Anal. Chem.* **88**, 4644–4650 (2016).
28. Samuel, A. Z., Horii, S., Ando, M. & Takeyama, H. Deconstruction of Obscure Features in SVD-Decomposed Raman Images from P. chrysogenum Reveals Complex Mixing of Spectra from Five Cellular Constituents. *Anal. Chem.* **93**, 12139–12146 (2021).
29. Pätzold, R. *et al.* In situ mapping of nitrifiers and anammox bacteria in microbial aggregates by means of confocal resonance Raman microscopy. *J. Microbiol. Methods* **72**, 241–248 (2008).
30. Lin, V. J. C. & Koenig, J. L. Raman studies of bovine serum albumin. *Biopolymers* **15**, 203–218 (1976).
