# Supplementary material for: Direct imaging of intracellular RNA, DNA, and liquid–liquid phase separated membraneless organelles with Raman microspectroscopy
Source: Commun Biol. 2022 Dec 17;5:1383. doi: 10.1038/s42003-022-04342-4 (PMC9759543; doi:10.1038/s42003-022-04342-4)
Supplement: Supplementary file 2 — Description of Additional Supplementary Files [file 42003_2022_4342_MOESM2_ESM.pdf]

Description of Additional Supplementary Files

File name: Supplementary Data 1

Description: The source data behind the graph Fig. S3 (box plot) in the paper
